# Hyper IgG4-Related Disease Presenting with Orbital Tumor and Immune Deficiency

**DOI:** 10.1155/2021/9260051

**Published:** 2021-09-18

**Authors:** Caroline G. Olson, Nancy Y. Olson

**Affiliations:** ^1^University of Missouri-Kansas City, School of Medicine, Kansas City, MO, USA; ^2^Allergy and Rheumatology Clinic of Kansas City, Overland Park, KS, USA

## Abstract

We report a case of IgG4-RD in a patient with high IgG4 levels, low functional antibodies, and low IgM levels. He presented with bilateral orbital pseudotumors and, after initial improvement on corticosteroids, relapsed with recurrent pleural effusion and pelvic pseudotumor. He had a grossly elevated serum IgG (1905 mg/dl) with elevations in all IgG subclasses but marked elevation in IgG4 (412 mg/dl), low IgM, and low pneumococcal antibodies. Orbital mass biopsy showed polyclonal lymphocytic infiltration and increased IgG4 plasma cells. The patient was started on prednisone and tried several immunosuppressive medications including mycophenolate mofetil, methotrexate, hydroxychloroquine, and azathioprine with decrease in size of the orbital pseudotumor. During a period when the patient stopped his medications, the pseudotumor enlarged with new development of recurrent pleural effusions. He was also found to have a pelvic mass that was biopsy positive for IgG4 proliferation. This case with progression to multiorgan involvement highlights the importance of identifying patients with IgG4-related disease. In contrast to previous cases with normal-to-high IgM, the IgM was low with impaired functional antibodies.

## 1. Introduction

Immunoglobulin G4-related disease (IgG4-RD) is a rare relapsing-remitting fibroinflammatory disease caused by monoclonal proliferation of IgG4+ plasma cells and dense lymphoplasmacytic infiltration with reversible collagen deposition [1–3]. It typically presents with insidious onset in middle-aged to elderly men, although studies have shown higher IgG4 levels and worse disease in Asian populations [1, 3–5]. IgG4+ plasma cells produce antibodies that may regulate the immune system and protect against hypersensitivity anaphylaxis in patients with allergies [6, 7]. Patients often have a longstanding history of allergic rhinitis, bronchial asthma, or eczema at diagnosis. IgG4- RD can be asymptomatic or present with symptoms of mechanical compression exerted by pseudotumor-like fibrotic masses. The most common manifestations have been grouped into four clinical phenotypes including pancreatobiliary (type 1 autoimmune pancreatitis (AIP)), retroperitoneal fibrosis and aortitis, head and neck limited, and systemic with salivary or lacrimal gland swelling (Mikulicz disease) [1, 8–10]. One study found that IgG4 played a role in all archival cases of idiopathic retroperitoneal fibrosis analyzed [4]. Atypical presentations including tubulointerstitial nephritis, glomerulonephritis, perineural disease, prostatitis, orchitis, sclerosing mastitis, eosinophilic angiocentric fibrosis, interstitial lung disease, and pleural and pericardial effusions have been reported [1, 11, 12]. Intrathoracic involvement can include parenchymal opacities and interstitial lung disease, tracheobronchial stenosis, fibrosing mediastinitis, nodular pleural lesions, and rarely, pleural effusion and may resemble sarcoidosis or lung cancer [3, 13]. Salivary and lacrimal gland involvement can cause facial and orbital swelling and can present similarly to Sjogren's syndrome with sclerosing sialadenitis [1, 14]. Orbital pseudotumors involve the lacrimal gland in 69% of cases but can also occur in extraocular muscles, palpebrae, optic nerve, and orbital bone, as well causing exophthalmos and restriction of ocular movements [1, 15–17]. These orbital manifestations tend to have higher relapse rates on steroids and overall have higher cumulative doses of corticosteroids [10]. Several studies have shown a link between lacrimal gland involvement and development of lymphoma [18]. The 2019 ACR/EULAR standards for diagnosis of IgG4-RD are based on the presence of characteristic clinical or radiological involvement of a typical organ or pathological evidence of an inflammatory lymphoplasmacytic infiltrate of uncertain etiology. In addition, the patients must not have another explanation for their symptoms including infection, autoimmune disease, or malignancy. Thirdly, there is a grading scale with points awarded for histopathology with dense lymphoplasmacytic infiltrate and storiform fibrosis with or without obliterative phlebitis, positive immunostaining, serum IgG4 >5x the upper limit of normal, having a set of glands involved, and having pulmonary or pancreas findings, kidney findings, and retroperitoneal findings [19]. In 10–20% of cases, antinuclear antibodies (ANAs) are also positive [4]. Cases of IgG4-RD have been described with elevated or normal levels of IgM, but low IgM and low functional antibodies have not previously been reported.

## 2. Case Presentation

The patient was a 66-year-old Caucasian male with class III obesity (BMI 42) and a history of adult-onset asthma and allergies referred by his ophthalmologist for bilateral orbital masses that showed IgG4 lymphoproliferative disease on biopsy. On the right side, the medial superior orbital mass was approximately 1.0 cm × 1.3 cm and left-sided mass was 4 mm × 4 mm. A biopsy of both right and left eyelid lesions including the bone and inferior orbital fat pad showed polyclonal lymphocytic infiltration and increased IgG4 plasma cells up to 98/HPF. The majority were T cells with a nonspecific mild increase in CD4:CD8 ratio and normal antigen expression. B cells were polyclonal with normal antigen expression. There was no immunophenotypic evidence of non-Hodgkin lymphoma. The diagnosis of IgG4 lymphoproliferative disease was given based on elevated IgG4 levels and pathologic findings, and he was referred to rheumatology. Systemic review was positive for fatigue, easy bruising and bleeding, cough, shortness of air, wheezing, hypertension, joint pain, red dry eyes, and postnasal drip. He did not smoke, denied recurrent infection other than rhinitis, and had no pertinent family history.

CT of the chest showed no evidence of pulmonary parenchymal fibrosis or interstitial lung disease. Laboratory investigations at the initial visit showed elevated IgG with extremely elevated IgG4, decreased functional antibodies, and low IgM. Total IgG 1905 mg/dl (694–1618 mg/dl), IgG1 1106 mg/dl (382–929 mg/dl), IgG2 598 mg/dl (241–700 mg/dl), IgG3 98 mg/dl (22–178 mg/dl), and IgG4 412 mg/dl (4–86 mg/dl). This gives an IgG4/IgG ratio of .216 which correlates with values ≥0.2 in patients with IgG4-RD. IgA was normal at 222, and IgM was 40. His initial pneumococcal vaccination was in three years prior to presentation. Three of 14 pneumococcal antibody titers were at a protective level (>1.3 mcg/ml). Haemophilus influenza antibodies were decreased at 0.34. Angiotensin-converting enzyme (ACE) was low. Rheumatoid factor was positive at 28, and cyclic citrullinated peptide (CCP) was negative.

**Figure 1:**
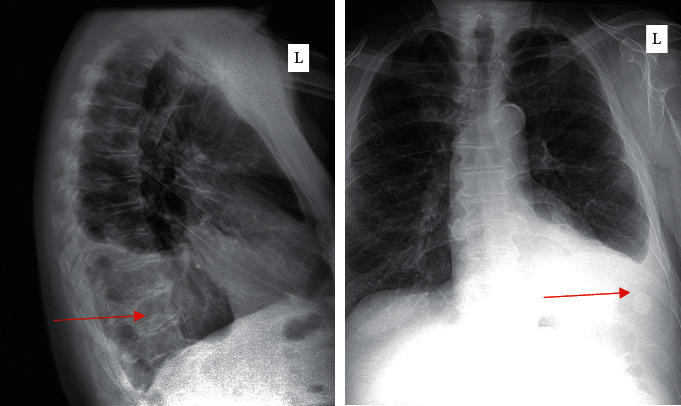
Lateral and posterior-anterior chest X-ray showing left-sided pleural effusion (arrows) during symptom relapse.

The patient was started on prednisone 20 mg daily, and to decrease steroid need with his weight, mycophenolate mofetil 1000 mg BID was added. Mycophenolate was discontinued due to side effects of dizziness and nausea. He was dissatisfied with steroid weight gain at prednisone 20 mg daily (BMI had increased to 44.9). On this regimen, he had marked improvement in asthma symptoms and reduced size of orbital pseudotumors. To continue to try to achieve corticosteroid sparing, he briefly used methotrexate and then added hydroxychloroquine as an adjunctive treatment with no further change in tumor size, so he was unwilling to continue them. Pneumovax booster and one Prevnar booster dose were administered while the patient was off immunosuppression. An additional Prevnar booster was given while he was on low-dose prednisone. He had an increase in pneumococcal protective antibodies from 3 of 14 to 4 of 14 during treatment. When the larger right orbital pseudotumor had regressed, the patient preferred to stop taking the prednisone and declined treatment for a period of 18 months. During this time, his bilateral eye lesions returned to near baseline size before treatment. Laboratory studies at this time showed an eosinophil count of 1096 (normal <500), SPEP with increased protein, decreased IgM at 20 mg/dl (normal >48 mg/dl), IgG 3570 mg/dl (normal <1618 mg/dl), IgG4 1594 mg/dl (normal <86 mg/dl), IgG4/IgG ratio of 0.45, ESR of 104 mm/h (normal <20 mm/h), and elevated CRP at 9.9 mg/dl (normal <8.0 mg/dl) (See Table 1). The patient also began to report increased shortness of breath, and chest X-ray showed large left pleural effusion (Figure 1). Thoracentesis yielded 2 L of fluid from the left lung that was negative for malignant cells but showed the presence of reactive mesothelial cells and histiocytes. He saw cardiology and had workup for pleural effusion including neoplastic and infectious causes.

The patient was unwilling to take corticosteroids again because of the efforts he had made to lose weight. He was willing to try azathioprine. He had a recurrence of left-sided pleural effusion, and repeat thoracentesis showed similar findings. He underwent pleurodesis on the left thorax. He was started on azathioprine 50 mg BID and corticosteroids, and during this period, the right orbital pseudotumor was at its smallest appearance. At this time, the patient was following with oncology and the CT/PET scan showed 3.3 cm × 1.9 cm mass in his pelvis near the left seminal vesicle as well as incidental liver lesion without PET scan uptake. Biopsy of the pelvic mass showed IgG4 stain strongly positive. Oncology started prednisone 80 mg daily with taper, and the size of the pelvic mass decreased in size to 2.1 cm × 1.1 cm. The patient continues to undergo ongoing treatment with intermittent steroids and azathioprine. Current pneumococcal antibody levels after a total of two Prevnar and two Pneumovax booster doses are 7 out of 14 protective.

## 3. Discussion

This patient presented with hyper IgG4-related orbital pseudotumors but, over time, developed recurrent pleural effusions and a pelvic pseudotumor with notable laboratory findings including low IgM and low functional protective antibodies.

The IgG4 subclass accounts for only 3–6% of the total IgG fraction, and up to 5% of the normal healthy population may have elevated IgG4 levels [3, 6, 19, 20]. IgG4 has been linked to several diseases that are clinically separate from IgG4-RD including pemphigus vulgaris, membranous glomerulonephritis, rheumatoid arthritis, Castleman syndrome, Wegener granulomatosis, pulmonary abscess, and thrombotic thrombocytopenic purpura [1, 21–23]. In IgG4-related disease, the hallmark is a lymphoplasmacytic infiltrate mainly consisting of IgG4+ plasma cells along with characteristic histological findings. The role of IgG4 plasma cells is not clearly understood as studies have not provided insight into this process. CD19, CD27, and CD38+ plasma blasts, as well as clonally expanded CD4+ and CD8+ cytotoxic T lymphocytes, have been found to be elevated in the blood and in fibrotic lesions of patients with IgG4-RD [2, 24, 25]. One study showed a significant presence of M2 macrophages among collagen fibers in fibrotic areas [26]. Cytokines thought to be involved in this process include IL-13 and TGF-B in fibroblast activation, as well as IL-4 and IL-10 in class switching of IgG to IgG4 [2]. A Japanese study has identified several loci that indicated increased susceptibility for development of IgG4-RD including HLA-DRB1 and FC-y [27]. Serological findings in IgG4-RD are nonspecific but can include elevations in erythrocyte sedimentation rate (ESR) and CRP, as well as positive ANA in 50% of patients and elevated RF in 20% [28]. Eosinophilia with increased IgE has been found in 30–50% of IgG4-RD lesions, and an eosinophilic predominance can be seen in orbital or upper respiratory tract disease [1, 21]. Typical serum protein electrophoresis shows polyclonal hypergammaglobulinemia with beta-gamma bridging [5]. Serum IgG4 levels above 1.35 g/dl may indicate higher risk for type 1 AIP and IgG4-related sclerosing cholangitis and can be used to differentiate pancreatic cancer from sclerosing cholangitis [20, 29, 30]. It may be useful to monitor serial IgG4 levels or circulating plasmablasts to guide treatment, but evidence is still unclear [1, 11, 31]. Two histological findings are needed for diagnosis including whorly storiform fibrosis, obliterative phlebitis, dense lymphoplasmacytic infiltrate with many IgG4+ plasma cells, and eosinophilic infiltrate or granuloma formation [1, 6, 17, 23].

In IgG4-related disease, IgM, IgA, and other IgG subclasses are frequently elevated [1]. In contrast to the current literature, our patient presented with persistently suppressed IgM levels and low protective antibody levels. In healthy individuals, IgM autoantibodies play an important role in modulating immune responses and clearing self-antigens [32–34]. IgM deficiency typically presents with repeated infections but may also have an increased rate of allergic and autoimmune disease than the general population [32, 34]. Almost 40% of patients with IgM deficiency present with asthma and allergic rhinitis [32]. One study showed that patients with IgM deficiency had a higher rate of pathogenic ANA and 14% developed autoimmune disease [35]. Hashimoto's thyroiditis, SLE, multiple myositis, myasthenia gravis, and Addison's disease have been linked to low IgM levels [32, 36]. Although this patient did not have significant sinopulmonary infections, he may have been at risk for autoimmune disease related to immune deficiency. In IgM deficiency, cases of IgG subclass deficiency have been reported, but hyper IgG4 disease has been reported only in one previous case report [34, 36, 37]. Immune response to vaccination has not been mentioned in IgG4-related disease. Some patients with selective IgM deficiency or IgG subclass deficiency have been shown to have impaired IgG responses to the pneumococcal polysaccharide vaccines which may improve with immunoglobulin therapy [34, 38]. At presentation, this patient was positive for only 3 of 14 protective pneumococcal antibodies as well as low haemophilus protective antibodies. His response to Pneumovax was poor. Repeated pneumococcal vaccines both Pneumovax and Prevnar were required to maintain his protective antibodies, and overall, he had a much weaker than expected polysaccharide response, only increasing from 3 to 4 protective antibodies after vaccination.

Treatment of IgG4-RD typically consists of prednisolone 0.6 mg/kg/d with gradual taper over a period of 3–6 months [39]. Patients with elevated IgG4 at baseline, multiorgan involvement, and history of disease relapse may have a higher tendency to relapse after steroids have been discontinued. As a result, glucocorticoid sparing treatments have been used including azathioprine, mycophenolate mofetil, methotrexate, cyclophosphamide, and tacrolimus, but studies have not been conducted to compare efficacies of these agents [39]. Glucocorticoids with immunosuppressants have been shown to have a higher relapse-free rate than glucocorticoids alone [16]. In patients with refractory disease, rituximab can decrease serum IgG4 subclass concentrations. Early treatment is more likely to improve disease manifestations in patients with prominent lymphoplasmacytic infiltrate and especially in glandular tissues [1, 40]. When disease progresses to tightly organized collagen bundles, definitive treatment is less successful, but potential to reverse collagen formation is still present. In this patient, the smallest tumor size was achieved with glucocorticoid taper. Glucocorticoids also caused weight gain, so the patient was reluctant to restart them after losing weight, so several glucocorticoid-sparing treatments were tried in conjunction, with the most symptom improvement during treatment with mycophenolate mofetil and azathioprine. His case was complicated by poor adherence to treatment due to medication side effects and lack of concern on his part once the orbital lesions decreased in size.

Several case studies have described incidence of immune thrombocytopenia in patients with IgG4 disease. The incidence of this finding is unknown but has been postulated to be caused by antiplatelet antibodies or other IgG subclasses of platelet antibody [41]. This patient had normal platelet levels throughout presentation and treatment. Isolated pleural effusion has been reported in several case studies of IgG4-related disease, but it is much more commonly seen with other lung manifestations such as parenchymal or pleural lesions [8, 42].

This case report highlights and demonstrates the relative effectiveness of steroid-sparing medications in IgG4-RD treatment, and shows a rare presentation of IgG4-RD with low IgM levels and low functional antibodies.

## Figures and Tables

**Table 1:** Laboratory values at presentation, several months into treatment while on corticosteroids and mycophenolate mofetil, and during relapse.

	IgG total (nl 694–1618 mg/dl)	IgG4 (nl 4–86 mg/dl)	IgM (nl >48 mg/dl)	ESR (nl <20 mm/hr)	CRP (nl <8 mg/dl)	Pneumococcal antibodies (nl >8/14)	Haemophilus antibodies (nl >1)	Platelets (nl 150–450)	Eosinophils (nl 15–500)
Presentation	1905	412	44	19	0.25	2/14	0.26	258	17
During treatment	1760	377	43	11	1.09	3/14	0.34	240	432
Relapse	3570	1594	20	104	9.3	4/14	0.17	250	1096

## Data Availability

No data were used to support this study other than patient medical record.
